# The Plasma Level of Interleukin-1β Can Be a Biomarker of Angiopathy in Systemic Chronic Active Epstein–Barr Virus Infection

**DOI:** 10.3389/fmicb.2022.874998

**Published:** 2022-04-06

**Authors:** Ayaka Ohashi, Yu Uemura, Mayumi Yoshimori, Naomi Wada, Ken-Ichi Imadome, Kazuo Yudo, Takatoshi Koyama, Norio Shimizu, Miwako Nishio, Ayako Arai

**Affiliations:** ^1^Department of Frontier Medicine, Institute of Medical Science, St. Marianna University School of Medicine, Kanagawa, Japan; ^2^Department of Laboratory Molecular Genetics of Hematology, Graduate School of Medical and Dental Sciences, Tokyo Medical and Dental University (TMDU), Tokyo, Japan; ^3^Division of Hematology and Oncology, Department of Internal Medicine, St. Marianna University School of Medicine, Kanagawa, Japan; ^4^Department of Hematological Therapeutics, Graduate School of Medical and Dental Sciences, Tokyo Medical and Dental University (TMDU), Tokyo, Japan; ^5^Department of Advanced Medicine for Viral Infections, National Center for Child Health and Development, Tokyo, Japan; ^6^Department of Hematology, Tokyo Medical and Dental University (TMDU), Tokyo, Japan; ^7^Center of Stem Cell and Regenerative Medicine, Advanced Multidisciplinary Research Cluster, Institute of Research, Tokyo Medical and Dental University (TMDU), Tokyo, Japan

**Keywords:** sCAEBV, angiopathy, IL-1β, monocytes, coagulation

## Abstract

Systemic chronic active Epstein–Barr virus infection (sCAEBV) is an EBV-positive T- or NK-cell neoplasm revealing persistent systemic inflammation. Twenty-five percent of sCAEBV patients accompany angiopathy. It is crucial to clarify the mechanisms of angiopathy development in sCAEBV because angiopathy is one of the main causes of death. Interleukin-1β (IL-1β) is reported to be involved in angiopathy onset. We investigated if IL-1β plays a role as the inducer of angiopathy of sCAEBV. We detected elevated IL-1β levels in four out of 17 sCAEBV patient’s plasma. Interestingly, three out of the four had clinically associated angiopathy. None of the other patients with undetectable level of IL-1β had angiopathy. In all patients with high plasma levels of IL-1β and vascular lesions, EBV-infected cells were CD4-positive T cells. In one patient with high plasma IL-1β, the level of *IL-1β* mRNA of the monocytes was 17.2 times higher than the level of the same patient’s EBV-infected cells in peripheral blood. In Ea.hy926 cells, which are the models of vascular endothelial cells, IL-1β inhibited the proliferation and induced the surface coagulation activity. IL-1β is a potent biomarker and a potent therapeutic target to treat sCAEBV accompanying angiopathy.

## Introduction

Chronic active Epstein–Barr virus infection (CAEBV), an intractable rare disease, is one of EBV-positive T- or NK-cell neoplasms. In CAEBV, EBV-infected T- or NK-cells proliferate clonally, infiltrate peripheral blood and tissues, and then cause multiple organ dysfunction. The WHO classification which was revised in 2017 defined CAEBV as an EBV-positive T- or NK-cell lymphoproliferative disease and described its two subtypes: systemic CAEBV (sCAEBV) accompanied by systemic symptoms and cutaneous CAEBV whose lesions are limited to the skin, of severe hypersensitivity to mosquito bite (sHMB) and hydroa vacciniforme (HV; Quintanilla-Martinez et al., 2017). According to previous reports, CAEBV is chemotherapy-resistant and allogeneic hematopoietic stem cell transplantation (HSCT) is the only promising curative treatment strategy (Kimura et al., 2012; Yonese et al., 2020).

Systemic chronic active Epstein–Barr virus infection has not only neoplastic but also inflammatory aspect. The paper on the Japanese nationwide survey of sCAEBV by Yonese et al. (2020) published in 2020 reported that sCAEBV patients have various symptoms associated with persistent inflammation: fever (85%), hepatosplenomegaly (70%), lymphadenopathy (53%), and others. Yonese et al. (2020) also reported that 25% of sCAEBV patients have angiopathy, such as aneurysm (9%), cardiac dysfunction (9%), and vasculitis (7%). Angiopathy can be a cause of organ failure and degrades patient’s quality of life. In addition, vasculitis is one of the factors to determine the existence of sCAEBV disease activities associated with poor outcomes after HSCT (Kimura et al., 2012). Thus, it is crucial to clarify the mechanism of angiopathy development to establish an effective treatment strategy for sCAEBV.

Inflammatory cytokines, such as interleukin-1β (IL-1β) and tumor necrosis factor-α (TNF-α), are reported to be involved in angiopathy onset. IL-1β is produced from immunocompetent cells, directly downregulates cell growth (Cozzolino et al., 1990), and induces apoptosis of vascular endothelial cells (Wang et al., 2015). In Kawasaki disease, which is a condition with systemic vasculitis, elevated serum levels of IL-1β reflect the status of clinical course (Maury et al., 1988). TNF-α depresses cell viability and provokes apoptosis of human vascular endothelial cells (Xia et al., 2006). There is also a report on the serum level of TNF-α elevated in Takayasu’s arteritis, a chronic inflammatory disease with damages in medium and large arteries (Gao et al., 2020). Therefore, TNF-α is reported to be a therapeutic target of Takayasu’s arteritis (Molloy et al., 2008; Mekinian et al., 2021). Thus, we hypothesized that IL-1β and TNF-α play some roles to induce vascular endothelial cell damage in sCAEBV with angiopathy. We previously pointed out that the concentration of TNF-α is elevated in serum of sCAEBV patients (Arai et al., 2012; Onozawa et al., 2017). However, there is no report on IL-1β concentration in sCAEBV patients to date. In this study, we investigated the plasma levels of IL-1β and TNF-α and their associations with clinical findings of sCAEBV patients who were diagnosed and treated in our institution.

## Materials and Methods

### Diagnosis of sCAEBV

The patients were diagnosed as sCAEBV when meeting all the following four conditions suggested by a research group of Measures against Intractable Diseases of the Ministry of Health, Labour and Welfare of Japan (Kimura et al., 2012; Yonese et al., 2020): (1) inflammation persisting for more than 3 months, (2) increasing EBV-DNA in peripheral blood or in diseased tissue, (3) EBV-infected T- or NK-cells, and (4) not applicable to other known diseases. The criteria conform with the definition of CAEBV in the WHO classification issued in 2017 (Quintanilla-Martinez et al., 2017).

### The Isolation of EBV-Infected Cells and Monocytes in sCAEBV Patients

The EBV-infected cells were isolated as described previously (Onozawa et al., 2018). In brief, the peripheral blood mononuclear cells (PBMCs) from patients were isolated by density gradient centrifugation using Lymphoprep™ (Abbott Diagnostics Technologies AS, Oslo, Norway) and sorted into CD4-, CD8-, or CD56-positive fractions by using antibody-conjugated magnetic beads (Miltenyi Biotec, Bergisch Gladbach, Germany). The primary monocytes were obtained by negative selection assay using Pan Monocyte Isolation Kit (Miltenyi Biotec).

### Detection of the Clonality

The clonal proliferation of EBV-infected cells was detected by Southern blotting for EBV-terminal repeat (Raab-Traub and Flynn, 1986).

### Definition of Disease Activity

Disease status was defined according to the previous reports (Yonese et al., 2020). Patients with active disease were defined as those with persistent findings of inflammation as follows: fever, liver dysfunction, progressive skin lesions, vasculitis, or uveitis accompanied by a significant increase in EBV-DNA. Liver dysfunction was defined as an increase in alanine transaminase levels to two times higher than the upper limit of normal. Progressive skin lesions and vasculitis were diagnosed by pathological examination. Uveitis was diagnosed by attending physicians and ophthalmologists.

### Quantification of Cytokines

Systemic chronic active Epstein–Barr virus infection patient’s plasma was collected by centrifugation. IL-1β and TNF-α in plasma were measured by high-sensitivity cytokine beads assay according to the manufacturer’s instructions (MILLIPLEX®MAP Kit, EMD Millipore Corporation, Massachusetts, United States). IL-18 in plasma was detected by U-PLEX Human IL-18 Antibody (Meso Scale Diagnostics, LLC., Maryland, United States).

### Cells and Reagents

Ea.hy926, a permanent Human umbilical vein endothelial cell (HUVEC) line, was used as a model of vascular endothelial cells kindly provided by Edgell et al. (1983). Human recombinant IL-1β was purchased from PeproTech (New Jersey, United States).

### Stimulation of Ea.hy926 Cells With IL-1β

Ea.hy926 cells were seeded and incubated for 24 h before stimulation. After 24 h, culture media were changed to serum-free media, and adhered cells were stimulated with IL-1β for 24 h. After stimulation for indicated time, the cells were collected for the following assays.

### XTT Assay

After stimulating Ea.hy926 cells with IL-1β for 24 h, the cell proliferation was analyzed by XTT assay according to the manufacturer’s instructions (Cell Count Reagent SF, nacalai tesque, Kyoto, Japan).

### qRT-PCR Analysis

RNA was extracted with ISOGEN II (Nippon Gene Co., Ltd., Tokyo, Japan). cDNA reactions were made by PrimeScript RT Master Mix (Takara Bio Inc., Shiga, Japan). qRT-PCR was performed on Light Cycler 480® (Roche Diagnostics International AG, Rotkreuz, Switzerland) using TaqMan® Gene Expression Assays (Thermo Fisher Scientific). We performed qRT-PCR to investigate mRNA expression of inflammatory cytokine, *IL-1β*, and coagulation-related factors, *tissue factor* (*TF*), *plasminogen activator inhibitor-1* (*PAI-1*), and *thrombomodulin* (*TM*). We used primers, *IL-1β* (Hs01555410_m1), *TF* (Hs01076029_m1), *PAI-1* (Hs00167155_m1), *TM* (Hs00264920_s1), and *GAPDH* (Hs99999905_m1; Thermo Fisher Scientific).

### Procoagulant Activity Assay

Interleukin-1β-stimulated Ea.hy926 cells were suspended in 50 μl of phosphate-buffered saline and added to 50 μl of pooled plasma from healthy donors. After incubation at 37°C for 3 min, 50 μl of 25 mM calcium chloride was added, and the plasma recalcification time was measured using a semi-automatic coagulator (CA-101, Sysmex, Hyogo, Japan; Koyama et al., 1994). The measurement of CA-101 was based on turbo-densitometry. Shortening of the coagulation time indicated the activation of clotting ability.

### Statistical Analysis

Data are presented as mean ± SD. Statistical analysis was processed using student’s two-tailed *t*-tests and Mann–Whitney test using GraphPad Prism 6 (GraphPad, California, United States). The significant differences are indicated by (*) for (*p* < 0.05), (**) for (*p* < 0.01), and (***) for (*p* < 0.001) compared to the control.

## Results

### The Concentrations of IL-1β and TNF-α in the Plasma of sCAEBV Patients

**Figure 1:**
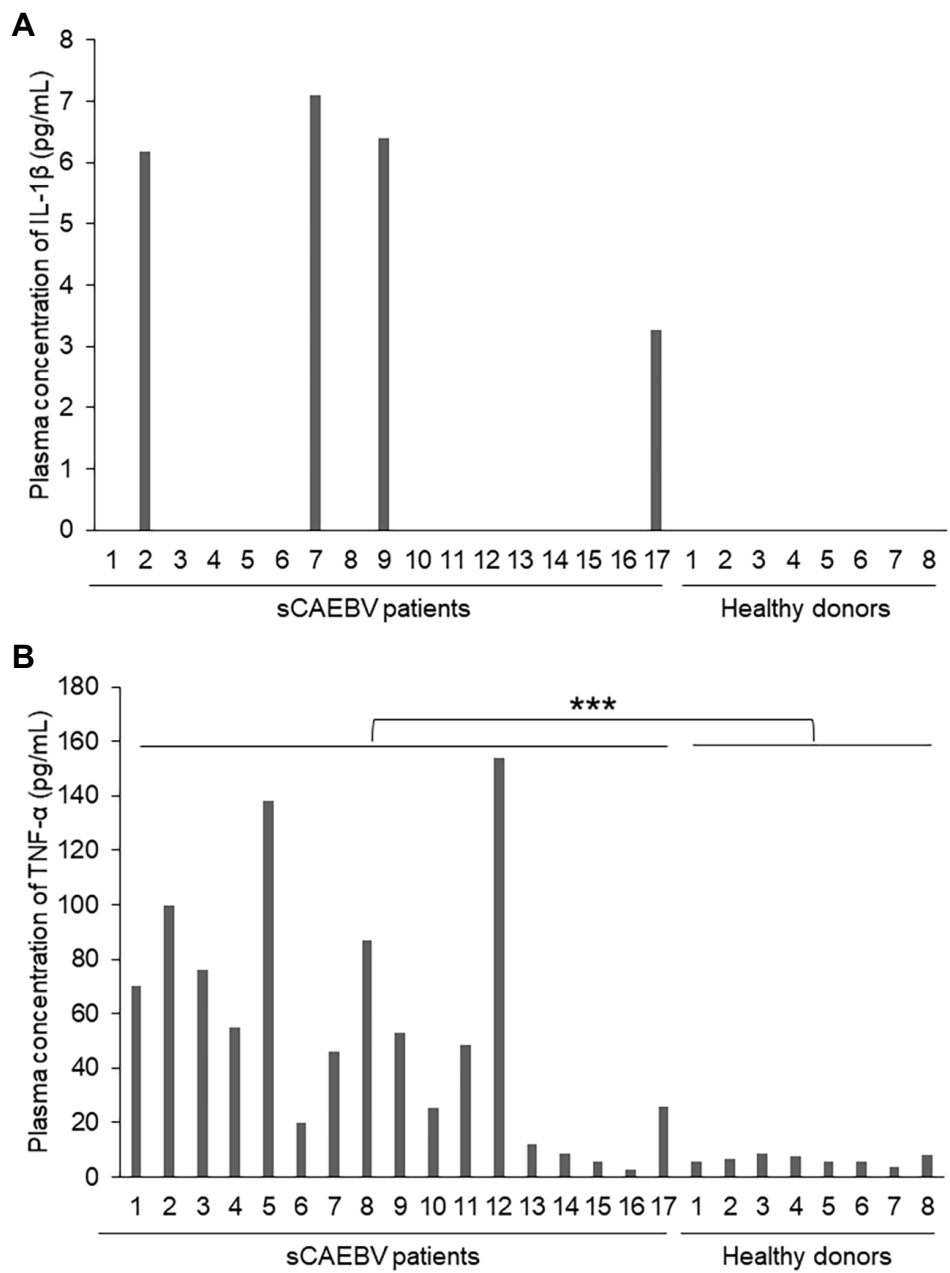
The concentrations of interleukin-1β (IL-1β) and tumor necrosis factor-α (TNF-α) in the plasma of Systemic chronic active Epstein–Barr virus infection (sCAEBV) patients. **(A)** IL-1β and **(B)** TNF-α concentrations in sCAEBV patients plasma measured by high-sensitivity cytokine beads assay. **(A)** IL-1β was detected only in patient 2, 7, 9, and 17. **(B)** The TNF-α concentration of patient’s plasma was significantly higher than that of healthy donors (^***^p < 0.001).

We used plasma samples from 17 sCAEBV patients whose ages were from 18 to 63 years. The characteristics of sCAEBV patients are shown in Table 1. Of the 17 patients, nine were male and eight were female. The types of EBV-infected cells were as follows: seven were CD4, one was CD8, and nine were CD56. The concentrations of IL-1β and TNF-α in the plasma from 17 sCAEBV patients and eight healthy donors were measured by high-sensitivity cytokine beads assay (Figures 1A,B). IL-1β levels were elevated in four out of 17 sCAEBV patient’s plasma. The levels in the plasma of the rest of the patients and the healthy donors were not detectable. Interestingly, among the four sCAEBV patients with elevated IL-1β, three had clinically diagnosed angiopathy, one had intracranial vascular lesion with multiple cerebral bleedings, and two had aneurysms. The EBV-infected cells of these three patients were CD4-positive cells. None of the other patients with undetectable level of IL-1β had angiopathy. The TNF-α concentration of patient’s plasma was significantly higher than that of healthy donors, but there was no correlation with angiopathy.

**Table 1:** Patient characteristics.

Patient no.	Gender	Age	EBV-Infected cells	Clonality of the EBV-infected cells	Clinical findings	Disease activity at the examination
1	M	22	CD4	Monoclonal	Fever, uveitis	A
2	M	36	CD4	Monoclonal	Fever, liver dysfunction, multiple cerebral and cerebellar micro bleedings	A
3	M	39	CD4	Monoclonal	Fever, colitis	I
4	F	21	CD56	Monoclonal	Fever, cytopenia	A
5	F	34	CD56	Monoclonal	Fever, cytopenia	A
6	M	33	CD4	Monoclonal	Fever	A
7	M	49	CD4	Monoclonal	Fever, hematemesis with right hyoid artery aneurysm	A
8	M	28	CD56	Monoclonal	Fever, cytopenia	A
9	F	22	CD4	Monoclonal	Fever, liver dysfunction, stenosis, and dilation of the vertebral artery	A
10	M	63	CD56	Monoclonal	Fever, cytopenia	A
11	M	47	CD56	Monoclonal	Fever, liver dysfunction	A
12	F	40	CD56	Monoclonal	Fever, liver dysfunction, and pulmonary hypertension	A
13	F	35	CD56	Monoclonal	Fever, cytopenia	A
14	F	30	CD4	Monoclonal	Fever, HV	I
15	M	42	CD56	Monoclonal	Fever, cytopenia	I
16	F	18	CD56	Monoclonal	Fever, sMBA	I
17	F	25	CD8	Monoclonal	Fever, liver dysfunction	A

F, female; M, male; HV, hydroa vacciniforme; sMBA, severe hypersensitivity to mosquito bite; A, active; and I, inactive.

### The Image Findings of the Vascular Lesions of sCAEBV Patients With Detectable Plasma Levels of IL-1β

**Figure 2:**
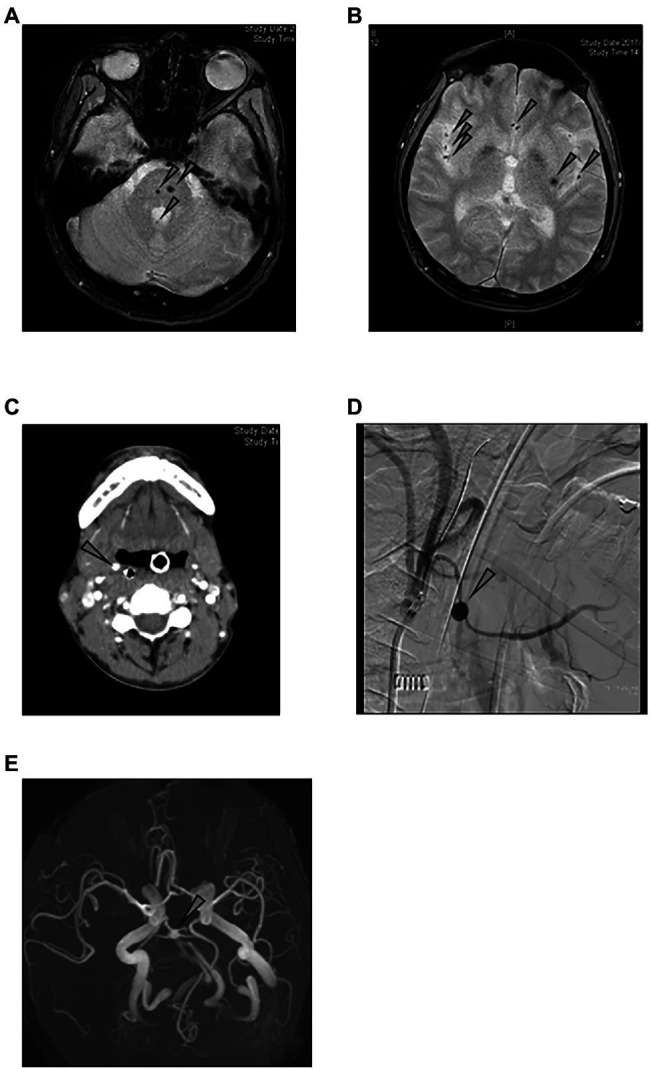
Image findings of sCAEBV patients who had angiopathy. **(A,B)** Magnetic resonance imaging of the brain of patient 2: Multiple cerebral **(A)** and cerebellar **(B)** micro bleeding were detected by T2 star-weighted images (arrows). **(C,D)** Images of patient 7: Contract-enhanced CT examination shows the dilatation of the right hyoid artery **(C)**. Angiography shows the right hyoid artery aneurysm **(D)**. **(E)** Magnetic resonance angiography of patient 9: Aneurysm was suspected because of narrowing and dilation of vertebral arteries.

Figure 2 shows the image findings of the vascular lesions of three patients with detectable plasma levels of IL-1β. Patient 2 was a 36-year-old male whose EBV-infected cell phenotype was CD4-positive T cells, and his disease activity was positive. Screening examination of the brain by magnetic resonance imaging at the diagnosis showed multiple micro bleedings (Figures 2A,B), and the presence of intracranial vascular lesions were assumed. Patient 7 was a 49-year-old male, whose EBV-infected cell phenotype was CD4-positive T cells, and his disease activity was positive. His main complaint at the diagnosis was hematemesis. Contrast-enhanced CT examination showed the dilatation of the right hyoid artery (Figure 2C). We confirmed a right hyoid artery aneurysm, which was the source of bleeding, by angiography (Figure 2D). Patient 9 was a 22-year-old female, whose EBV-infected cell phenotype was also CD4, harboring active disease. Although she had no neurological symptom, magnetic resonance angiography at the diagnosis showed narrowing and the dilation of the vertebral artery, which led us to speculate aneurysm (Figure 2E).

### *IL-1β* mRNA Expression in EBV-Infected Cells and Monocytes in sCAEBV Patients With Detectable Plasma Levels of IL-1β

**Figure 3:**
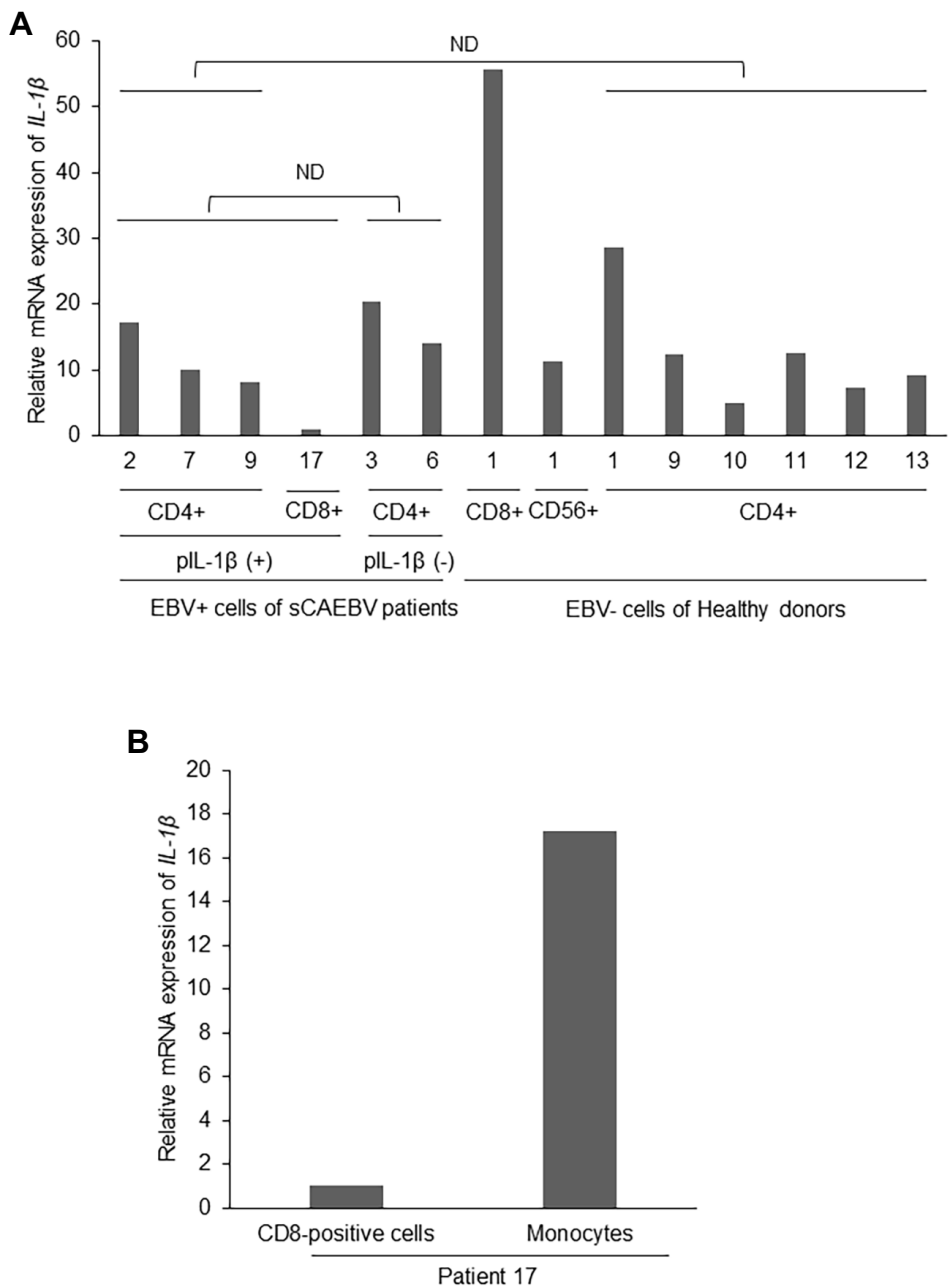
*Interleukin-1β* mRNA expression in EBV-infected cells of sCAEBV patients. **(A)** EBV-infected cells were collected from patients 2 (CD4), 3 (CD4), 6 (CD4), 7 (CD4), 9 (CD4), and 17 (CD8). Patient 2, 7, 9, and 17 had IL-1β in their plasma while patient 3 and 6 had no plasma IL-1β. CD4-positive cells were collected from six healthy donors and CD8- and CD56-positive cells were from the same healthy donor. The mRNA expression of *IL-1β* in EBV-infected cells was analyzed by qRT-PCR assay. The expression was normalized to *GAPDH* mRNA. The value of vertical axis showed the expression in CD8-positive cells of patient 17 as 1. There were no significant differences between *IL-1β* mRNA expression in the EBV-positive cells of patients with high plasma IL-1β and patients with undetectable level of plasma IL-1β. The mRNA expression of *IL-1β* in the EBV-positive cells of sCAEBV patients was not higher than that of the same lymphocyte fractions of healthy donors. The statistical comparison was performed for mRNA of CD4-positive cells. **(B)** CD8-positive cells and monocytes derived from patient 17, who had plasma IL-1β. The mRNA expression of *IL-1β* in EBV-infected cells was analyzed by qRT-PCR assay. The expression was normalized to *GAPDH* mRNA. The value of vertical axis showed the expression in CD8-positive cells of patient 17 as 1. ND: no significant difference. pIL-1β: plasma IL-1β.

We searched for the cells that produced IL-1β in sCAEBV. We first focused on EBV-infected cells. We divided the patients into two groups: a group with detectable plasma IL-1β formulated by patient 2, 7, 9, and 17, and a group of undetectable plasma IL-1β formulated by patients 3 and 6. We compared the mRNA expression of *IL-1β* in EBV-positive T- or NK-cells isolated from peripheral blood of the groups. As shown in Figure 3A, there was no statistical difference of *IL-1β* mRNA between two groups. Next, we compared *IL-1β* mRNA expression of the patients with that of healthy donors. There was no difference of *IL-1β* mRNA of the patients’ EBV-infected cells and that of the same lymphocyte fractions of healthy donors. These results suggest that EBV-infected cells were not the main producers of IL-1β.

We then focused on monocytes which may be producing IL-1β. In patient 17, who had detectable plasma level of IL-1β, the level of *IL-1β* mRNA of monocytes isolated from peripheral blood was higher than the level of the same patient’s EBV-infected cells (Figure 3B). This result suggests that monocytes produced IL-1β.

### The Concentration of IL-18 in the Plasma of sCAEBV Patients With Detectable Plasma Levels of IL-1β

**Figure 4:**
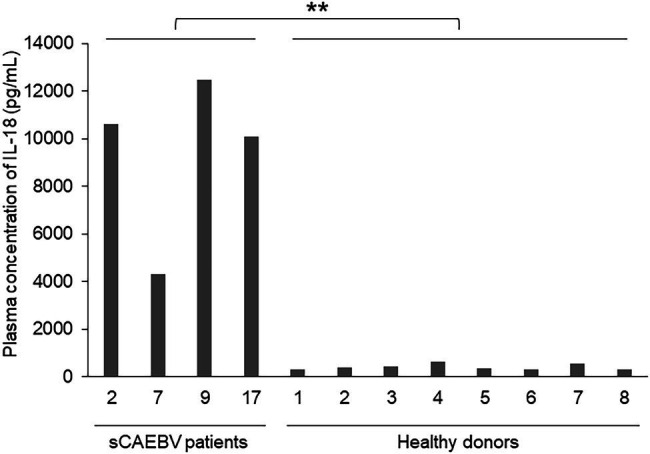
The concentrations of IL-18 in the plasma of sCAEBV patients. The concentrations of IL-18 in the plasma of four sCAEBV patients and eight healthy donors were measured by U-PLEX Human IL-18 Antibody. There were significant differences between IL-18 plasma concentration in patients with plasma IL-1β and healthy donors (^**^p < 0.01).

It is known that the protein complex called inflammasome is largely involved in the production of IL-1β. Inflammasome is activated by the invasion of pathogen through pattern recognition receptors, which contribute to develop inflammation. Therefore, we examined if IL-1β were produced in inflammasomes of sCAEBV patients. IL-1β and IL-18 are inflammatory cytokines, and both are produced in the inflammasomes of immunocompetent cells by caspase-1-induced cleavage from their precursors. Figure 4 shows the plasma levels of IL-18 in sCAEBV patients, who had detectable plasma level of IL-1β, and in healthy donors. IL-18 concentration in patients was higher than in healthy donors. The result suggested that both IL-1β and IL-18 are produced in the inflammasomes of sCAEBV patients following the same procedures.

### The *in vitro* Effects of IL-1β on a Vascular Endothelial Cell Line Ea.hy926 Cells

**Figure 5:**
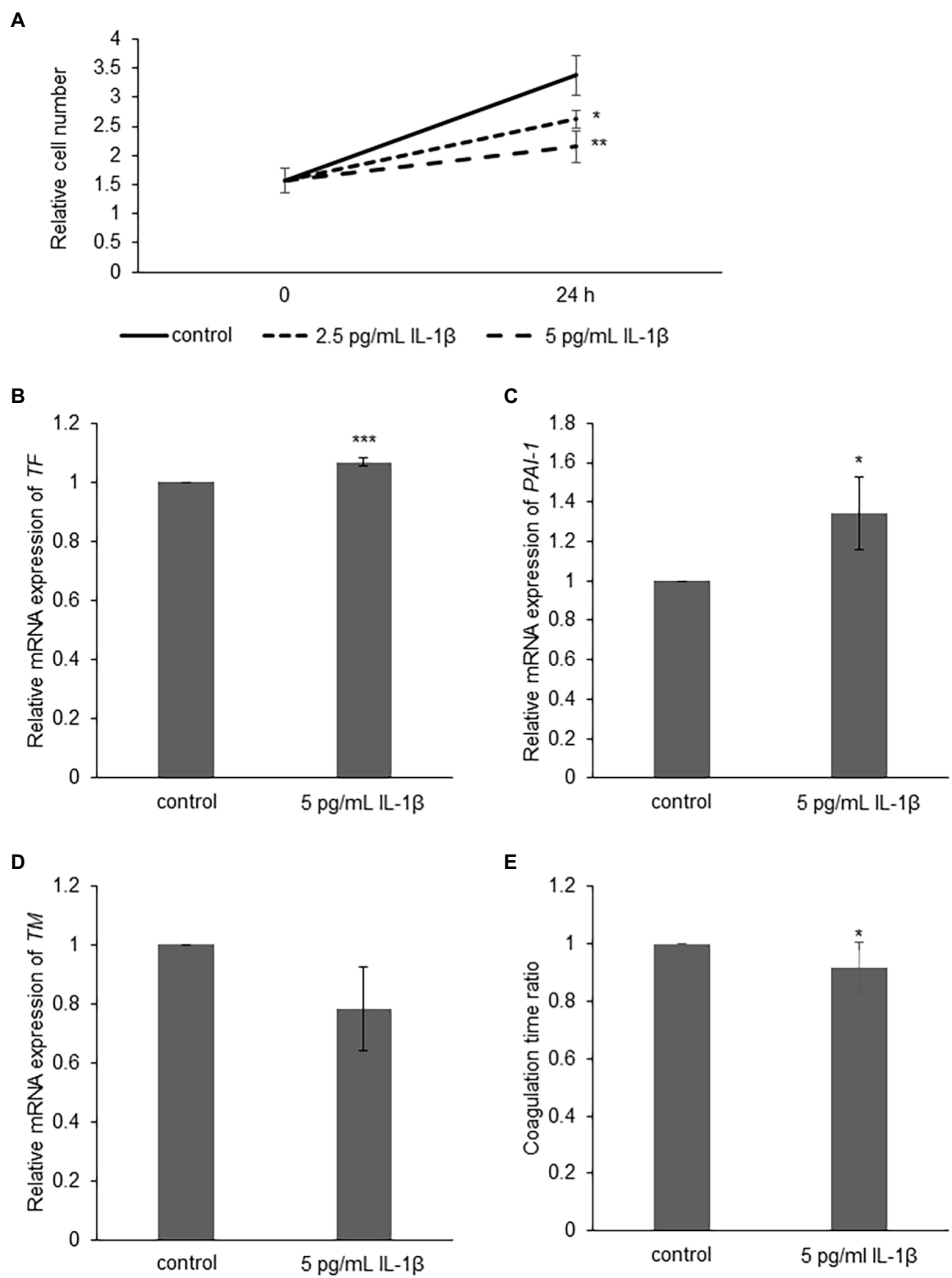
The effect of IL-1β on Ea.hy926 cells. **(A)** Ea.hy926 cells were treated with 2.5 and 5 pg/ml of IL-1β for 24 h. After the treatment, the proliferation of Ea.hy926 cells was examined by XTT assay. The data are shown as mean ± SD (*n* = 3). Significant differences are indicated as * for *p* < 0.05 and ** for *p* < 0.01 between control and others at 24 h time point. **(B-D)** Ea.hy926 cells were treated with 5 pg/ml of IL-1β for 24 h. After the treatment, the cells were harvested for qRT-PCR assay. **(B)** The mRNA expression of *tissue factor* (*TF*), **(C)** mRNA expression of *Plasminogen activator inhibitor-1* (*PAI-1*), and **(D)** *thrombomodulin* (*TM*) were analyzed by qRT-PCR assay. The expression was normalized to *GAPDH* mRNA. The data are shown as mean ± SD (*n* = 3). Significant differences are indicated as * for *p* < 0.05 and *** for *p* < 0.001 between control (showed as 1) and stimulated one. **(E)** Ea.hy926 cells were treated with 5 pg/ml of IL-1β for 24 h. After the treatment, the cells were harvested for procoagulant activity (PCA) assay. The PCA on Ea.hy926 cells was measured by normal plasma-based recalcification time. The shortening of coagulation time indicates increased PCA. The value of vertical axis showed the coagulation time of control as 1. The data are shown as mean ± SD (*n* = 5). Significant differences are indicated as * for *p* < 0.05 between control and stimulated one.

To examine the role of IL-1β in the development of angiopathy in sCAEBV, we investigated the direct effects of IL-1β on blood vessels. We added IL-1β directly into the culture medium of a vascular endothelial cell line, Ea.hy926 cells, and analyzed their viable cell numbers by XTT assay. IL-1β of the concentrations equivalent to the concentrations in the patients inhibited the viable cell number of Ea.hy926 cells (Figure 5A). Next, we investigated the effects of IL-1β on molecules contributing to vascular damage and blood coagulation. We stimulated vascular endothelial cells with IL-1β and measured the mRNA expression of coagulation-related factors by qRT-PCR analysis. IL-1β upregulated the mRNA expression of *TF* as well as *PAI-1* (Figures 5B,C) and suppressed the mRNA of *TM* (Figure 5D) in Ea.hy926 cells. Finally, we analyzed if IL-1β activates the surface clotting ability of Ea.hy926 cells by procoagulant activity (PCA) assay. IL-1β reduced coagulation time, which meant that IL-1β caused coagulation activity (Figure 5E).

## Discussion

Interleukin-1β was detected in the plasma of all three sCAEBV patients with vascular lesions analyzed in this study, while none of the other patients with undetectable level of IL-1β had angiopathy. IL-1β caused vascular endothelial cells to suppress the proliferation and enhance surface coagulation activity *in vitro*. We suspected that IL-1β could be one of the causes of angiopathy onset.

All patients with high plasma levels of IL-1β and vascular lesions were CD4 infected type. However, the number of the examined samples was too small to clarify if there is a unique IL-1β production mechanism in CAEBV with CD4-positive cells. In our study, there was no difference in *IL-1β* mRNA expression in EBV-infected cells between the patients with high plasma level of IL-1β and the patients with undetectable plasma IL-1β. This suggests that IL-1β may be produced by cells other than EBV-infected cells. However, it is reported that IL-1β originally exist in the form of precursor protein (pro-IL-1β) in immunocompetent cells, such as macrophage and CD4-positive cells (Brough and Rothwell, 2007; Pulugulla et al., 2018). During viral infection, pro-IL-1β is cleaved by inflammasome-derived caspase-1 and become a mature product (Yu and Finlay, 2008). Therefore, the production of IL-1β can be induced without mRNA elevation (Pulugulla et al., 2018). It is known that IL-18 is produced by the same mechanism as IL-1β. In the cases of which IL-1β was detected in plasma, the plasma level of IL-18 also elevated. In these cases, inflammasome-derived caspase-1 may be activated, and we can assume that IL-1β and IL-18 were produced as a result. We had reported our earlier discovery of NF-κB constitutively activated in EBV-positive T- or NK-cells in CAEBV (Takada et al., 2017). NF-κB enhances the expression of pro-IL-1β, which is called “priming,” and NLRP3, which is a constituent of inflammasome (Bauernfeind et al., 2009). Furthermore, Chen et al. (2012) reported that EBV directly induced IL-1β induction through the activation of inflammasome in EBV-associated nasopharyngeal carcinoma cells. We need to further explore the mechanisms of inflammasome activation and IL-1β production in EBV-positive T- or NK-cells.

*Interleukin-1β* mRNA expression in the monocytes of patient 17, whose IL-1β was detected in plasma, was higher than the expression of EBV-positive T cells. Monocyte is another possible cell producing IL-1β in sCAEBV. Recently, myeloid derived suppressor cells (MDSCs) were detected in the peripheral blood of sCAEBV patients (Collins et al., 2021). MDSCs are immature bone marrow cells and have a strong immunosuppressive effect. In patients of psoriasis, a disease with chronic systemic inflammation, MDSCs produce *IL-1β* (Cao et al., 2016). Therefore, we suspect that MDSCs of sCAEBV patients may also produce IL-1β. We need to further validate the existence of IL-1β-producing cells in sCAEBV.

We investigated the possibility of IL-1β directly damaging vascular endothelial cells. IL-1β in the concentration similar to that in patient plasma suppressed the viable cell number of vascular endothelial cells. IL-1β is active in picomolar concentrations (Chin et al., 1988). In inflammatory lesions of CAEBV patients, the concentration may be locally elevated, and excessive IL-1β may be causing apoptosis of vascular endothelial cells. In the same cells, IL-1β also induced *TF* and *PAI-1* expression, suppressed *TM* expression, and then enhanced procoagulation activity. These results suggest that IL-1β may directly induce vascular damage and blood coagulation, which can lead to angiopathy.

We must identify the reason why IL-1β level is high only in certain sCAEBV patients. It is reported that single nucleotide polymorphisms (SNPs) of IL-1β, rs16944, and rs1143627, are related to IL-1β serum levels in patients of febrile seizure (Choi et al., 2019) and antisynthetase syndrome (Ponce-Gallegos et al., 2020). Interestingly, these SNPs contribute to increasing the risk of coronary artery lesions in Kawasaki disease (Fu et al., 2019). However, the mutations of rs16944: A > G, rs1143627: G > A, which are specific to only in the patients with vascular lesions, were not found (data not shown) in 10 sCAEBV patients including two harboring vascular lesions. The number of cases was insufficient to determine the association with known SNPs in this analysis. We speculate that the cause of high IL-1β plasma concentration in some patients is related to multiple factors, such as the infectious type of EBV-infected cells and SNPs. We need to investigate in a larger number of cases in the future.

The limitation of this study is small number of the samples due to rarity of the disease. We were able to collect only 17 patient samples. To prove our proposal hypothesis, we need more clinical specimens to verify and investigate also *in vivo*.

Our results suggest that IL-1β may be a therapeutic target to treat sCAEBV accompanying angiopathy. Anakinra, an IL-1β antagonist, is used as a therapeutic agent for hemophagocytic lymphohistiocytosis (HLH; Bami et al., 2020; Kavirayani et al., 2020). Anakinra is also used to treat COVID-19 (Filocamo et al., 2020) because the macrophage NLRP3 inflammasome is activated and IL-1β production is promoted (Chen et al., 2019). The combination of anakinra and ruxolitinib is reported beneficial to the disease (Kaplanski et al., 2021). We see a hope in these medications to be applied for the treatment of sCAEBV with vascular lesions. All three patients with high plasma IL-1β and angiopathy had passed away. We will continue to pay attention to IL-1β as a pathological condition indicator and a therapeutic target. In conclusion, IL-1β may be a biomarker and a therapeutic target to treat sCAEBV accompanying vascular lesions.

## Data Availability Statement

The original contributions presented in the study are included in the article/supplementary material, further inquiries can be directed to the corresponding author.

## Ethics Statement

The studies involving human participants were reviewed and approved by the ethical committees of St. Marianna University School of Medicine and Tokyo Medical and Dental University. The patients/participants provided their written informed consent to participate in this study.

## Author Contributions

AO and AA designed the research, performed the experiments, analyzed the data, and wrote the draft. YU, MY, NW, and K-II performed the experiments and analyzed the data. KY, TK, NS, and MN analyzed the data. AA collected the samples. All authors contributed to the article and approved the submitted version.

## Funding

This research was funded by Japan Agency for Medical Research and Development (AMED), as their “Practical Research Projects for Rare/Intractable Diseases (18ek0109334h0001, 19ek0109334h0002, 20ek0109334h0003, and 21ek0109334h0004)” and by Japan Society for the Promotion of Science under the grant programs “Grant-in-Aid for Early-Career Scientists (20K16410)” and “Grant-in-Aid for Research Activity Start-up (19K23907).”

## Conflict of Interest

The authors declare that the research was conducted in the absence of any commercial or financial relationships that could be construed as a potential conflict of interest.

## Publisher’s Note

All claims expressed in this article are solely those of the authors and do not necessarily represent those of their affiliated organizations, or those of the publisher, the editors and the reviewers. Any product that may be evaluated in this article, or claim that may be made by its manufacturer, is not guaranteed or endorsed by the publisher.
